# Supplementary material for: Thousands of high-quality sequencing samples fail to show meaningful correlation between 5S and 45S ribosomal DNA arrays in humans
Source: Sci Rep. 2021 Jan 11;11:449. doi: 10.1038/s41598-020-80049-y (PMC7801704; doi:10.1038/s41598-020-80049-y)
Supplement: Supplementary file 4 — Supplementary Information 4. [file 41598_2020_80049_MOESM4_ESM.pdf]

Thousands of high-quality sequencing samples fail to show meaningful correlation between 5S and 45S ribosomal DNA arrays in humans

Ashley N. Hall[1,2], Tychele N. Turner[3*], Christine Queitsch[1*]

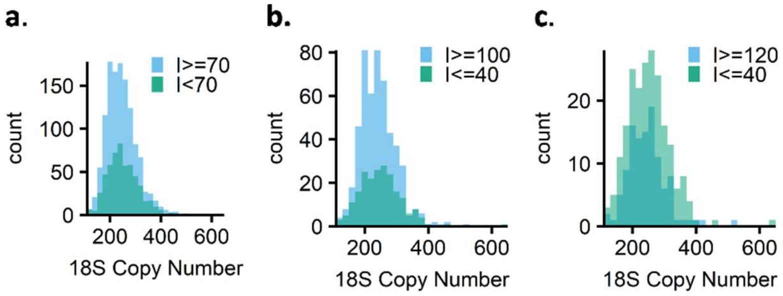

Figure S1: Additional comparisons of rDNA copy number of probands with differing IQ. a: Comparison of rDNA copy number in probands at the cutoff of pathological IQ (I<70, n=548 compared to I>=70, n=1,244). Welch Two Sample t-test p-value: 0.03. **b:** Comparison of rDNA copy number of probands with IQ<=40 (n=210) and IQ>=100 (n=500). Welch Two Sample t-test p-value: 0.1103. **c:** Comparison of rDNA copy number of probands with a more stringent IQ cutoff: I<=40 (n=210) compared to I>=120 (n=112). Welch Two Sample t-test p-value: 0.4129.

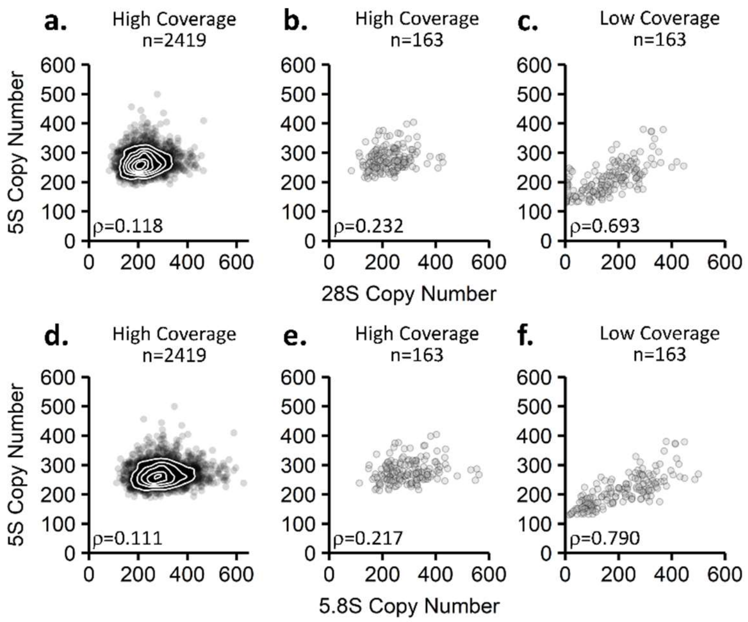

Figure S2: Correlations of rDNA copy numbers in 1000 Genomes Project data. A-C: Comparison of the 28S to 5S rDNA copy numbers in the (A) high-coverage 1000 Genomes Project data (n=2,419), (B) subset of high-coverage 1000 Genomes Project data also analyzed in the low-coverage dataset (n=163), and (C) low-coverage 1000 Genomes Project data (n=163). Bottom: Comparison of the 5.8S to the 5S rDNA copy numbers in the (D) high-coverage 1000 Genomes Project data (n=2,419), (E) subset of high-coverage 1000 Genomes Project data also analyzed in the low-coverage dataset (n=163), and (F) low-coverage 1000 Genomes Project data (n=163).

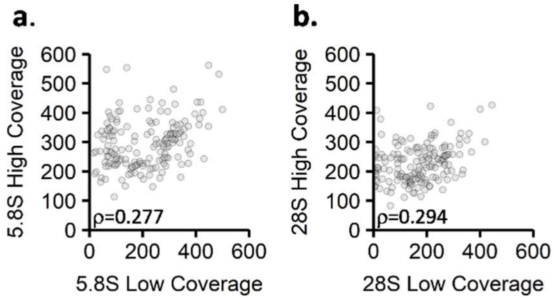

Figure S3: Replicability of 5.8S and 28S rDNA copy number estimates between the high- and low-coverage 1000 Genomes Project data. a: Comparison of 5.8S estimates between high- and low-coverage datasets (n=163). **b**: Comparison of 28S estimates between high- and low- coverage datasets (n=163).

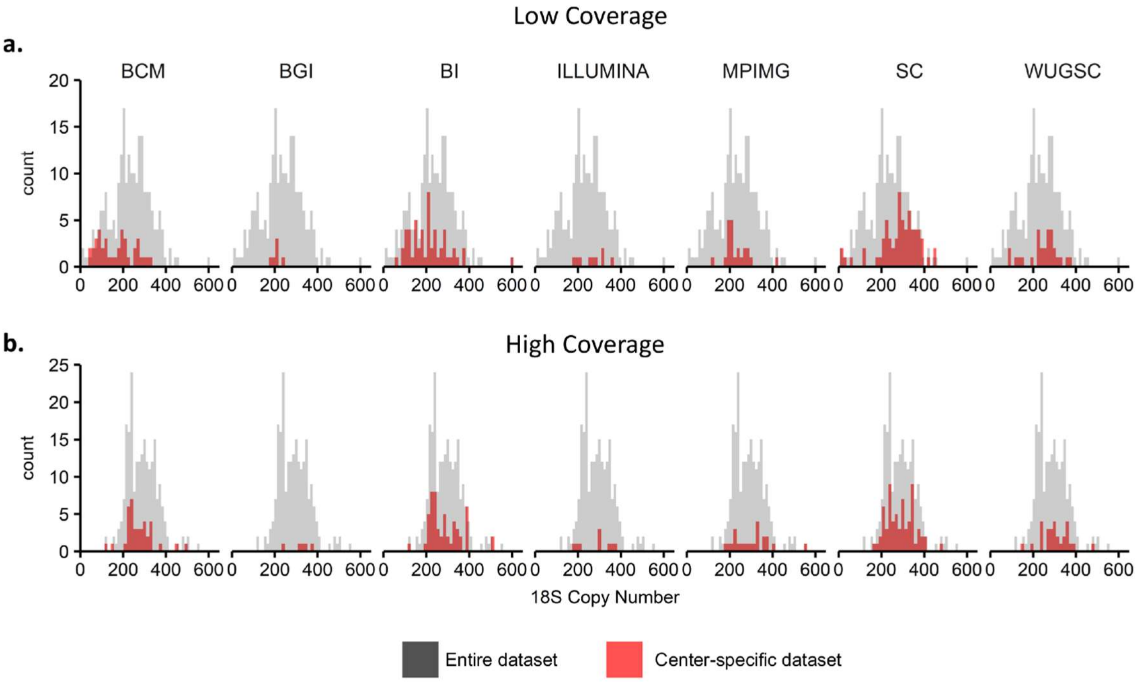

Figure S4: Distribution of 18S copy number estimates by sequencing center. a: Distribution of 18S copy number estimates from individual libraries sequenced by each of 7 sequencing centers (gray) for the 163 samples analyzed in the low coverage data (n=222 distinct libraries). Copy number estimates for samples produced by a given center in the low-coverage sequencing are highlighted in red. **b**: Distribution of 18S copy number estimates from the high-coverage 1000 Genomes Project data for the 163 samples analyzed in the low coverage data. The high-coverage data were not generated by any of these 7 centers: '**b**' serves to demonstrate whether a center was assigned samples with a higher or lower copy number distribution. Samples that were composed of multiple distinct libraries in the low-coverage data are represented multiple times in the high-coverage plot.

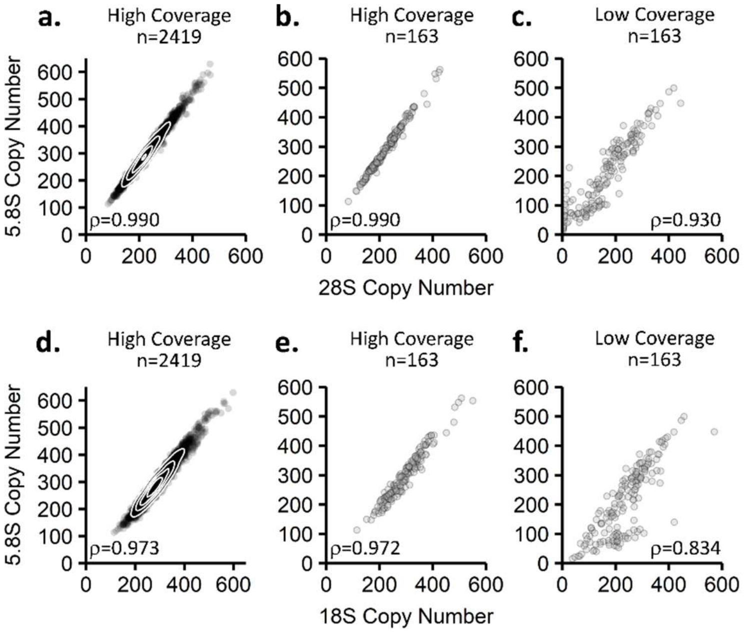

Figure S5: Comparison of copy number estimates of regions of the 45S rDNA repeat to each other. a-c: Comparison of the 28S to 5.8S rDNA copy numbers in the 1000 Genomes Project datasets. **a**: High-coverage 1000 Genomes Project data (n=2,419). **b**: Subset of high-coverage 1000 Genomes Project data also analyzed in the low-coverage dataset (n=163). **c**: Low-coverage 1000 Genomes Project data (n=163). **d-f**: Comparison of the 18S to the 5.8S rDNA copy numbers in the 1000 Genomes Project datasets. **d**: High-coverage 1000 Genomes Project data (n=2,419). **e**: Subset of high-coverage 1000 Genomes Project data also analyzed in the low-coverage dataset (n=163). **f**: Low-coverage 1000 Genomes Project data (n=163)

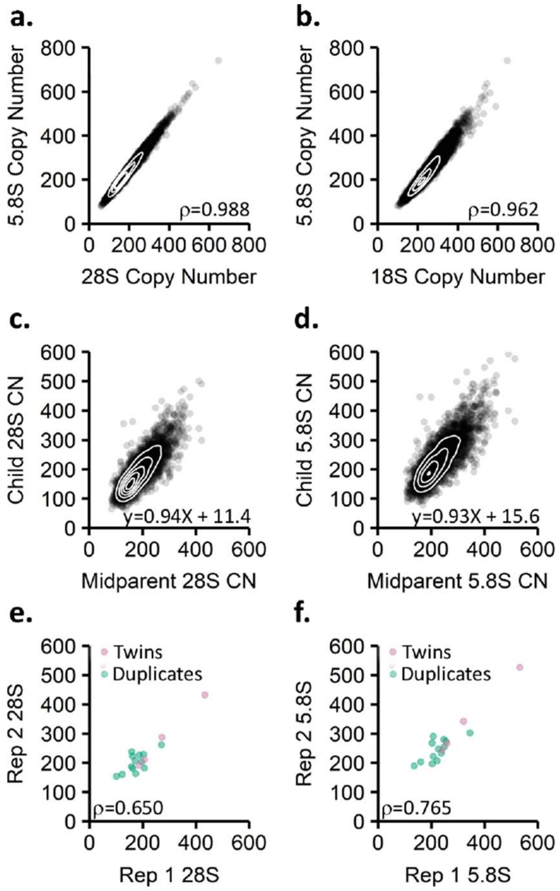

Figure S6: Data quality metrics for the Simons Simplex Collection. a: Comparison of the 28S to 5.8S rDNA copy numbers (n=7,210). **b**: Comparison of the 18S to 5.8S rDNA copy numbers (n=7,210). **c**: Heritability estimate of the 28S rDNA region (n=3,548). **d**: Heritability estimate of the 5.8S rDNA region (n=3,548). **e-f**: Comparison of 5.8S (**e**) and 28S (**f**) copy number estimates for either monozygotic twins (n=4 pairs) or for individuals sequenced twice in the Simons Simplex Collection (n=13). Spearman correlation indicated is for monozygotic twins and duplicates analyzed together.
